# Genetic Determinants of Paget’s Disease of Bone

**DOI:** 10.1007/s11914-021-00676-w

**Published:** 2021-05-14

**Authors:** Navnit S. Makaram, Stuart H. Ralston

**Affiliations:** Centre for Genomic and Experimental Medicine, Institute of Genetics and Cancer, University of Edinburgh, Edinburgh, EH4 2XU UK

**Keywords:** Paget's disease of bone, genetics, osteoclast, SQSTM1, multisystem proteinopathy

## Abstract

**Purpose of Review:**

To provide an overview of the role of genes and loci that predispose to Paget’s disease of bone and related disorders.

**Recent Findings:**

Studies over the past ten years have seen major advances in knowledge on the role of genetic factors in Paget’s disease of bone (PDB). Genome wide association studies have identified six loci that predispose to the disease whereas family based studies have identified a further eight genes that cause PDB. This brings the total number of genes and loci implicated in PDB to fourteen. Emerging evidence has shown that a number of these genes also predispose to multisystem proteinopathy syndromes where PDB is accompanied by neurodegeneration and myopathy due to the accumulation of abnormal protein aggregates, emphasising the importance of defects in autophagy in the pathogenesis of PDB.

**Summary:**

Genetic factors play a key role in the pathogenesis of PDB and the studies in this area have identified several genes previously not suspected to play a role in bone metabolism. Genetic testing coupled to targeted therapeutic intervention is being explored as a way of halting disease progression and improving outcome before irreversible skeletal damage has occurred.

## Introduction

Paget’s disease of bone (PDB) is a common skeletal disorder, characterised by focal regions of abnormal and disorganised bone remodelling at one (monostotic) or more (polyostotic) sites. The disease mainly involves the axial skeleton, such that the pelvis (70%), femur (55%), lumbar spine (53%), skull (42%), and tibia (32%) are preferentially affected. Clinically, the disease result in symptoms of bone pain, deformity, and pathological fracture. Osteoarthritis related to bone deformity and subchondral sclerosis is a common consequence and frequently requires arthroplasty [1]. Patients with PDB also have an increased risk of developing osteosarcoma, and although rare (0.3% of PDB patients), virtually all osteosarcomas that occur in adulthood do so in patients with PDB [2]. Similarly, some families have been described in which PDB is accompanied by giant cell tumours [3].

## Epidemiology

Paget’s disease of bone (PDB) is estimated to affect about 2% of the UK population over 55 years of age, with a greater incidence in men (1.4:1) [2]. There is marked geographical variation in the occurrence of PDB. The UK has the highest incidence of PDB in the world, but the disease is also common in other European countries such as Spain, Italy, and France. It is also common in people of European descent, and because of this, the incidence is high in countries that have historically been common destinations for migration of these communities, such as Australia, Canada, New Zealand, South Africa, and the USA [4, 5]. In contrast, PDB is rare in the Indian subcontinent, Scandinavia, China, and the Far East [6].

The incidence of PDB increases markedly with age; it is rare below the age of 50 years, but doubles each decade thereafter affect about 7.6% of men and 5.4% of women by the eighth decade in the UK [7]. The striking geographic variation in PDB and correlation with trends in the migration of certain population cohorts has emphasised the importance of genetic factors in the pathogenesis of PDB. Environmental factors also play a significant role however, reflected by the reduced incidence and severity of PDB in several locations including the UK, continental Europe, and New Zealand [2, 8, 9], but no significant change in other regions such as Italy and the USA [10–12]. The identity of environmental triggers for PDB is unclear [13]. Full discussion of this area is beyond the scope of this article, but suggested environmental influences include low dietary calcium intake [14], vitamin D deficiency [15], excessive biomechanical loading [16], tobacco use [17], wood heating during childhood [18], lead exposure [19, 20], air pollution [21], contact with animals [22], and a slow virus infection with one of the paramyxoviruses [23, 24].

## Inheritance of Paget’s Disease

Familial clustering is common in classical PDB, and in some families the disease is inherited in an autosomal dominant manner. In the UK, a family history of PDB is obtained in around 15% of PDB cases but in other regions such as Quebec, a positive family history is obtained in up to 50% of individuals [25]. It has been estimated that penetrance reaches 80–90% by the seventh decade in people who carry mutations in *SQSTM1* which is the most important predisposing gene for PDB [26–28] (see below). The relative risk of developing PDB in relatives of an affected individual is approximately seven times greater than in relatives of controls, and this rises to 20 times for relatives of patients with severe or early-onset disease [29]. Traditionally PDB is characterised into familial or sporadic subtypes, based on the presence of a positive family history, but it is important to remember that as the disease may be asymptomatic, family history may be more common than one thinks.

A number of rare syndromes have been identified in which clinical, radiological, or histological features in common with PDB are observed but where there is an early age at onset and the diseases are inherited in a Mendelian manner. The clinical and pathophysiological features of these disorders have recently been reviewed elsewhere [30•], but the genes responsible will also be discussed here.

A significant observation from recent findings has been the relatively large effect sizes of the loci influencing the development of PDB compared with other common diseases of the musculoskeletal system, such as osteoporosis and rheumatoid arthritis [31••]. This indicates that susceptibility to PDB is likely to be mediated by the inheritance of a relatively small number of genes with large effect sizes as opposed to a large number of genes with small effect sizes. For example, a number of common variants have been identified within or close to genes such as *CSF1*, *TNFRSF11A*, *TM7SF4*, *NUP205*, and *RIN3* which individually are not sufficient to cause the disease but which act cumulatively to significantly increase the risk of developing PDB.

## Candidate Genes for Paget’s Disease

**Fig. 1:**
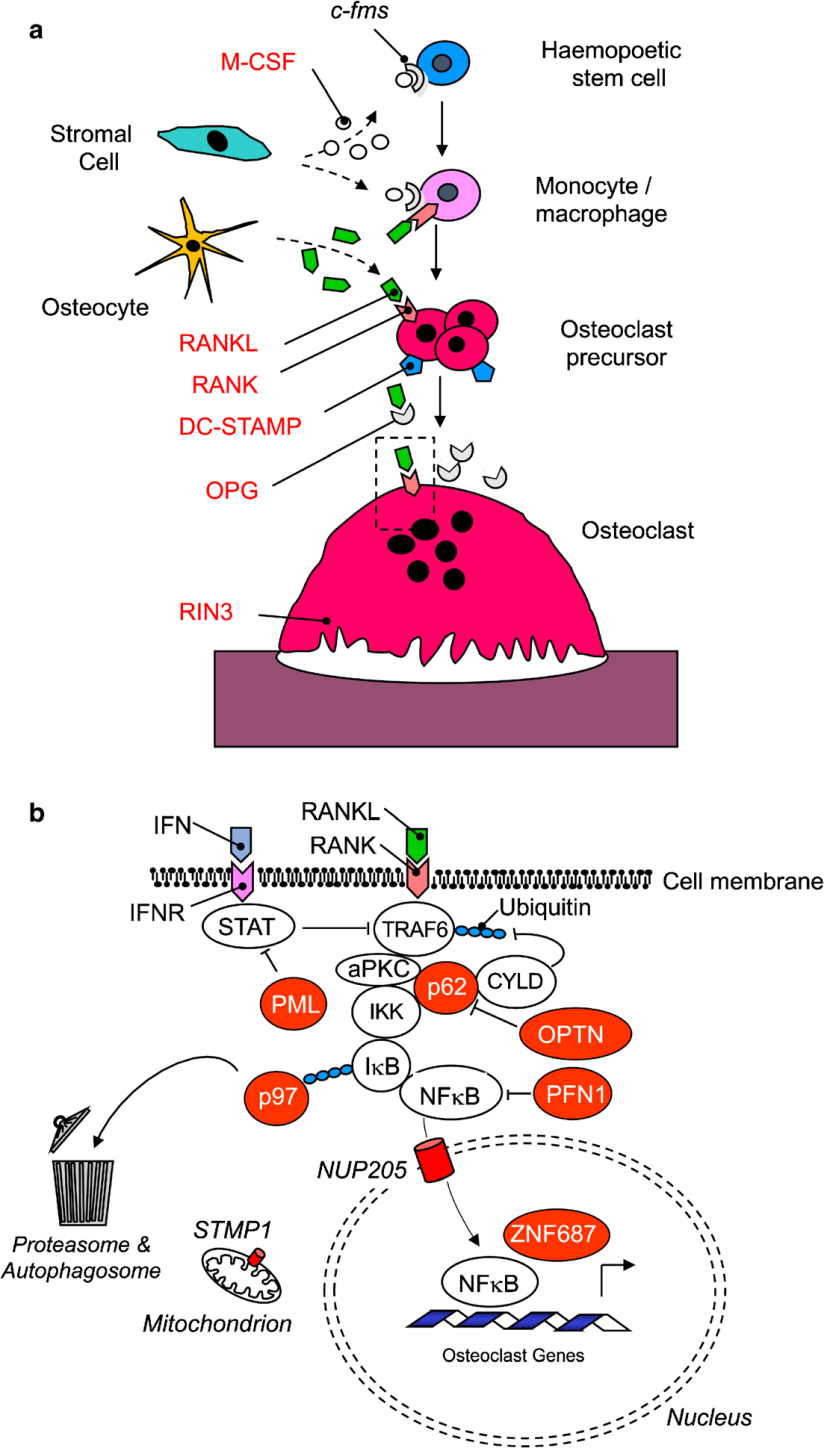
Genes implicated in Paget’s disease and related syndromes. Panel **a** shows some genes implicated in the pathogenesis of PDB which have effects on osteoclast differentiation and bone resorption. The gene products implicated in PDB are highlighted in red text. Panel **b** shows genes thought to affect intracellular signalling in osteoclasts and osteoclast precursors. The implicated gene products are highlighted in red and mostly affect the NFκB signalling pathway by interacting with *SQSTM1* or genes that regulate signalling downstream of the RANK receptor. The ZNF678 gene encodes a transcription factor, but the exact mechanisms by which it predisposes to PDB are unclear. Abbreviations: c-fms - Colony Stimulating Factor 1 Receptor; M-CSF - Macrophage Colony Stimulating factor; RANK - Receptor Activator of Nuclear Factor Kappa B; RANKL - RANK Ligand; DC-STAMP - Dendritic Cell Specific Transmembrane Protein; OPG - Osteoprotegerin; RIN3 - Ras and Rab Interactor 3; IFN - Interferon; IFNR - Interferon Receptor; STAT - Signal Transducers and Activators of Transcription; TRAF 6 - Tumour Necrosis Factor Receptor Associated Factor 6; CYLD - Cylindromatosis; p62 - Sequestosome 1; aPKC - atypical Protein Kinase C; IKK - Inhibitor of Kappa B Kinase; IkB - Inhibitor of Kappa B; NFκB - Nuclear Factor Kappa B; OPTN - Optineurin, PFN1 - Profilin 1; NUP205 - Nucleoporin 205; ZNF687 - Zinc Finger Protein 687; Short Transmembrane Mitochondrial Protein 1.

Several genes and loci that predispose to familial PDB have been identified over the last two decades by a combination of linkage analysis in families and genome wide association studies (GWAS) in unrelated individuals. Many of the PDB-associated genes have important roles in osteoclast differentiation and function as illustrated in Fig. 1.

### Colony Stimulating Factor 1

Variants at the colony stimulating factor *1 (CSF1)* locus on chromosome 1p13 were identified as predisposing to PDB by a GWAS study in 2010 [31••] and replicated in a further GWAS study [32••] more recently. The *CSF1* gene encodes macrophage-colony stimulating factor (M-CSF). This protein has a critical role in osteoclast formation and survival and is a strong functional candidate for PDB susceptibility [33]. The M-CSF protein binds to its receptor (c-FMS), resulting in a signalling cascade which leads to the activation of ERK (extracellular signal-regulated kinase) and AKT (protein kinase B), activating genes that promote the proliferation and survival of osteoclast precursors. Clinical studies have shown that patients with PDB have increased serum levels of M-CSF, supporting the role of *CSF1* in the pathogenesis of PDB [34]. The single nucleotide polymorphisms (SNPs) associated with PDB are located upstream of the gene in a region rich in regulatory elements, suggesting that the association may be due to an effect on regulation of *CSF1* expression but the exact mechanisms by which these variants predispose to PDB remain to be established.

### Heterogeneous Nuclear Ribonucleoproteins

Mutations in the heterogeneous nuclear ribonucleoprotein A2/B1 (*hnRNPA2B1)* and *hnRNPA1* genes were identified as a cause of multisystem proteinopathy in which PDB was a component part by a genome sequencing approach in families [35••]. The disease-causing mutations increase the propensity of the proteins to assemble into fibrils and to be recruited into cytoplasmic ‘stress granules’ (reviewed by Ralston and Taylor [30•]). It is of interest that a family has been described in which a pP310L mutation in *hnRNPA2B1* occurred in patients with PDB who did not experience myopathy or neurodegeneration [36•].

### Nucleoporin 205

The nucleoporin 205 *(NUP205)* gene on chromosome 7q33 was identified as possible candidate for PDB by an extended GWAS in 2011 [32••]. The strongest signal was with the rs4294134 within intron 22 of the gene. The NUP205 protein is involved in the nuclear pore complex (NPC) which is responsible for transport of molecules from the cytoplasm to the nucleus. The role of NUP205 in bone is currently unclear, but a previous study demonstrated that inactivating missense mutations in NUP205 are associated with steroid-resistant nephrotic syndrome [37, 38]. Another gene within the 7q33 locus that may be a stronger candidate is *STMP1* which is also located within the 7q33 locus. Mullin and colleagues recently reported that rs4294134 was an expression quantitative trait locus for *STMP1* which was found to be expressed in a human osteoclast specific cDNA library [39•]. The *STMP1* gene encodes a short transmembrane mitochondrial protein whose function in bone is unclear.

### Optineurin

The optineurin gene (*OPTN*) on chromosome 10p13 was identified as a candidate for PDB by linkage studies in families [40] and GWAS studies [31••, 32••]. The strongest association in GWAS was observed with the rs1561570 variant within the *OPTN* gene which increased the risk of PDB by about 1.6-fold. The *OPTN* gene encodes optineurin, which is a widely expressed cytoplasmic protein with multiple cellular functions, including in NFκB signalling [41], autophagy, and innate immunity [42]. Previous studies have identified a negative regulatory role for *OPTN* in osteoclast differentiation by modulating NFκB and interferon signalling both in vitro and *in vivo* in mouse models [43]. It has been shown that *OPTN* appears to negatively regulate RANKL-induced NFκB activation in osteoclast precursors by a mechanism requiring ubiquitin binding and involves an interaction with CYLD [43]. Knockdown of optineurin in bone marrow-derived macrophages was found to enhanced osteoclast differentiation and hypernucleation [43]. Furthermore, mice with a loss of function mutation in *Optn* exhibited enhance bone turnover and increased sensitivity of osteoclast precursors to RANKL stimulation [43]. The predisposing variant in humans is associated with significantly reduced *OPTN* mRNA expression. This indicates that *OPTN* is a negative regulator of osteoclast differentiation and function and that the predisposing variant in humans reduces *OPTN* expression, thereby increasing osteoclast activity.

### Promyelocytic Leukaemia Gene

The promyelocytic leukaemia gene *(PML)* on 15q24.1 was identified as a candidate for PDB following a GWAS study in 2011 [32••]. Although the locus contains two genes, *PML* was the most obvious candidate. The *PML* gene gained its name as it was discovered as a tumour suppressor that is disrupted in acute promyelocytic leukaemia resulting in its fusion to the gene retinoic acid receptor alpha (*RARA*) [44]. It has also been shown to be involved in multiple other cellular pathways, such as in regulation of cell growth, senescence, apoptosis, DNA repair, stem cell maintenance, protein degradation, autophagy, and antiviral responses [45]. Furthermore, it appears to maintain genome integrity and stability through recognition of DNA damage and organisation of multiple DNA repair complexes [46, 47].

Until recently the role of *PML* in bone metabolism was unknown, but previous studies suggested that it may regulate myeloid cell differentiation, proliferation, and osteogenic differentiation of mesenchymal stem cells [48, 49]. Recent preliminary studies published in abstract form have shown that PDB patients have reduced levels of *PML* expression compared with controls, and *in vitro* and *in vivo* studies have revealed that decreased expression of *PML* leads to increased osteoclast differentiation and activity [50]. Although the mechanisms by which *PML* variants predispose to PDB in humans are incompletely understood, it seems likely that they do so by increasing osteoclast differentiation and function.

### Profilin 1

A frameshift mutation in the profilin 1 gene *(PFN1)* was identified as a cause of severe early-onset PDB by a genome sequencing approach in a large family from Campania in Italy [51••]. Interestingly, three members of the family also developed osteosarcoma at a relatively young age. The causal mutation was predicted to produce a truncated variant of the gene product profilin-1. Functional studies showed that the abnormal protein increased osteoclast formation and multinuclearity *in vitro* in RAW273 cells and that the truncated protein also formed abnormal aggregates when expressed in a neuroblastoma cell line [51••]. Further studies also demonstrated evidence for loss-of-heterozygosity for the *PFN1* gene in about 2% of patients with sporadic PDB from the same region of Italy. It is of interest that previously, mutations of *PFN1* had been identified by other investigators as a cause of amyotrophic lateral sclerosis (ALS) and that when the abnormal proteins were studied *in vitro*, insoluble protein aggregates were formed [52]. From these observations, it would appear that *PFN1-*mediated PDB may represent an example of multisystem proteinopathy, recently coined as the subtype ‘multisystem proteinopathy 7 (MSP7)’ [53].

### Ras and Rab Interactor 3

The Ras and Rab interactor *(RIN3)* gene on chromosome 14q33 was identified as a candidate for PDB by extended GWAS. The strongest signal was tagged by the SNP rs10498635 within the gene encoding *RIN3*. The *RIN3* gene encodes Rab and Ras interactor protein 3, which acts as a guanine nucleotide exchange factor (GEF) for the Rab5 family of proteins including Rab5 itself and RAb31 [54]. Members of the RIN family interact with various proteins through functional domains they share, playing a role in endocytosis, vesicular trafficking, and signal transduction. These proteins are known to have a role in regulating osteoclast function through their effects on vesicular trafficking, but until recently, the role of *RIN3* in bone metabolism had not been specifically studied [55]. Functional studies have shown that *RIN3* mRNA is expressed in bone tissue and its expression level is approximately 10-fold higher in osteoclasts compared with osteoblasts [56]. The same authors also detected Rin3 protein in human osteoclasts from bone sections of a patient with giant cell tumour of bone, [56]. Functional studies have shown that Rin3^−/−^ mice have increased bone mass and reduced osteoclastic bone resorption *in vivo* but that osteoclast differentiation and survival *in vitro* are normal [57]. From this it was concluded that likely variants in RIN3 predispose to PDB through a gain-in-function. Interestingly Pavlos et al. [58] found a similar phenotype in mice with inactivation of the small GTPase Rab3D (a potential target for RIN3), raising the possibility that RIN3 may act as a GEF not only for the Rab5 sub-family of GTPases as has previously been reported [58–59] but also for Rab3D [59]. Having said that, further investigation is required to evaluate the specific pathway by which variants in *RIN3* produce their observed phenotypic effects.

### Transcription Factor SP7

The transcription factor *SP7* gene (SP7) was identified by Whyte and colleagues as a potential cause of early-onset PDB in a patient with an osteosclerotic disorder characterised by high bone mass, bone deformity, and pathological fractures during childhood. The patient had a raised serum ALP, hypertelorism, and a broad forehead with prominence of the frontal bone. There were bowing deformities of both femora. The clinical features were felt to be consistent with juvenile PDB [60•]. Genetic analysis revealed a heterozygous mutation at codon 309 (pS309T) causing a substitution of tryptophan for serine in a conserved region of the *SP7* gene product, Osterix. Histological analysis showed evidence of woven bone, but this did not show the characteristic features of increased osteoclastic bone resorption that is usual in PDB. The *SP7* gene encodes Osterix, a transcription factor essential for osteoblast differentiation. Loss of function mutations in SP7 are a recognised cause of osteogenesis imperfecta, and this is thought to occur as the result of reduced bone formation. It therefore seems likely that the pS309T mutation causes gain of function of Osterix and works by increasing bone formation, leading to production of woven bone and associated bone deformity and increased bone fragility.

### Sequestosome 1

Mutations in the sequestosome 1 *(SQSTM1)* gene were identified by genome wide linkage studies as a cause of familial PDB in two independent populations [27, 28]. Researchers in Quebec identified a proline to leucine mutation at codon 392 of the protein (P392L) as a cause of the disease [61], and soon after, this and other mutations were identified in the ubiquitin-associated (UBA) domain as the cause of the disease in people of UK descent [62]. A large number of *SQSTM1* mutations have now been reported in PDB patients across the world, most of which affect the UBA domain [61–65]. The frequency with which *SQTSM1* mutations occur in PDB varies in different countries, but in the UK, it has been estimated that they occur in about 40% of familial PDB cases and up to 10% of sporadic cases [25, 66]. The *SQSTM1* gene encodes p62, an adaptor protein in the NFκB signalling pathway [67]. This protein has a role in regulation of signalling downstream of the RANK, tumour necrosis factor (TNF), and neurotrophic growth factor (NGF) receptors [68] but also appears to play a key role in regulating other cellular processes through its involvement in autophagy [69–71]. This has recently been shown to be dysregulated in preclinical models of *SQSTM1*-mediated PDB [72] and by the finding of associations between SNP of genes in the autophagy pathway and PDB [73].

The mechanisms by which mutations in *SQSTM1* lead to PDB are not yet fully understood, but a common feature is their interference in the ability of p62 to bind ubiquitin [74]. This leads to enhanced NFκB signalling and increased sensitivity of osteoclast precursors to RANK-ligand (RANKL).

### Transmembrane 7 Superfamily Member 4

The transmembrane 7 superfamily member 4 *(TM7SF4)* gene on chromosome 8q22 encodes a seven-pass transmembrane protein that is primarily expressed in dendritic cells. It was identified as being associated with PDB by an extended GWAS in 2011 [32••]. The strongest association was within an 18-kb linkage disequilibrium block spanning the *TM7SF4* gene. There is a strong functional rationale for this being a candidate gene for PDB since it encodes a dendritic cell-specific transmembrane protein (DC-STAMP) which is upregulated by RANKL in osteoclast precursors [75] where it is required for fusion of osteoclast precursors to form mature osteoclasts [76]. A potential ligand for DC-STAMP is connective tissue growth factor (CCN2) which results in stimulation of osteoclastogenesis [77].

The variant identified by GWAS is also an expression quantitative trait locus for expression of *TM7SF4* in peripheral blood monocytes and osteoclasts raising the possibility that variants at this locus predispose to PDB by upregulating *TM7SF4* expression, thereby enhancing osteoclast fusion [39•, 78]. Some rare variants have also been identified in the protein coding region of *TM7SF4* in patients with PDB, but it is unclear what effects they have on protein function.

### Tumour Necrosis Factor Receptor Superfamily Member 11A

The tumour necrosis factor receptor superfamily member 11A *(TNFSRF11A)* gene on chromosome 18q21 encodes receptor activator of nuclear factor kappa B (RANK) which is a signalling molecule that plays an essential role in osteoclast differentiation and function. The *TNFRSF11A* gene was first implicated in the pathogenesis of PDB through linkage studies followed by positional cloning in familial expansile osteolysis (FEO) and early-onset familial Paget’s disease which identified mutations affecting the first exon of RANK as the cause of both disorders [79]. Subsequently similar mutations were identified as the cause of expansile skeletal hyperphosphatasia and panostotic expansile bone disease [30•]. The responsible mutations are duplications of between 15 and 27 nucleotides in the first exon of *TNFRSF11A* which add between five and nine additional amino acid residues into the RANK signal peptide, preventing its cleavage [79]. The abnormal RANK molecules accumulate in the Golgi apparatus [80] and promote osteoclast differentiation, probably by activating the unfolded protein response. Linkage studies in classical PDB also showed linkage to the same region of 18q21 with genetic heterogeneity [81] leading to investigations of the role of *TNFRSF11A* in classical PDB [82]. An association was observed with rs1805034 which codes for an alanine to valine amino acid change at codon 192 (pA192V). A significant association between pA192V and PDB was also reported in a case control study in an Italian population [83]. The mechanism by which the pA192V polymorphism predisposes to PDB is unclear since *in silico* analysis has predicted it to be a conservative change and functional studies have not showed differences in the ability of the two variants to stimulate NFκB activation *in vitro* [82]. The candidacy of *TNFRSF11A* as a predisposing gene for classical PDB was confirmed by a genome wide association study which showed association between PDB and the rs2957128 and rs3018362 variants in the 3′ flank of the TNFRSF11A gene [31••]. These variants are in moderate linkage disequilibrium with the pV192A variant, but at the present time, the molecular mechanisms by which *TNFRSF11A* variants predispose to PDB are incompletely understood.

### Tumour Necrosis Family Receptor Superfamily Member 11B

Loss of function mutations in the tumour necrosis family receptor superfamily member 11B gene *(TNFRSF11B)* were identified as a cause of the syndrome of juvenile PDB. This is a rare recessive disorder associated with grossly abnormal bone remodelling, bone expansion, and bone deformity presenting in childhood and adolescence. It is caused by various loss of function mutations affecting *TNFRSF11B* [84] which encodes osteoprotegerin (OPG), an inhibitor of osteoclast differentiation and function [84, 85]. Mutations of *TNFRSF11B* have not yet been detected in classical PDB, but there is some evidence from association studies to suggest that common variants at the *TNFRSF11B* locus may predispose to PDB in women but not men [86, 87].

### Valosin-Containing Protein

Mutations in the valosin-containing protein *(VCP)* gene were identified as the cause of the syndrome of hereditary inclusion body myopathy, PDB, and frontotemporal dementia (IBMPFD) [88]. The *VCP* gene encodes p97 which is a multifunctional protein involved in several intracellular processes including NFκB signalling, DNA repair, and autophagy. The mechanisms of neurological dysfunction and myopathy are incompletely understood but are likely to involve a general defect in ubiquitin mediated protein degradation with accumulation of abnormal protein aggregates which cause cellular toxicity. The PDB component is less well understood but may be due at least in part to increased sequestration of IκB with activation of NFκB. Since its original description, the IBMPFD syndrome associated with *VCP* mutations has been reclassified as one of the multisystem proteinopathies, which are multisystem disorders in which neurological and muscle dysfunction are sometimes accompanied by PDB [89].

### Zinc Finger Protein 687

Mutations in the zinc finger protein 687 *(ZNF687)* gene were identified as a cause of severe early-onset PDB by a genome sequencing approach in a large family with autosomal dominant inheritance of PDB from the Campania region of Italy [90••]. The PDB was accompanied by giant cell tumour (CGT) in 4/14 (28.5%) of affected patients from this family. The causal mutation in the index family was a proline to arginine amino acid substitution at codon 937 (pP937R) of *ZNF687*. Subsequently sequencing of other families from the same region identified the same mutation and another mutation which causes a serine to isoleucine amino change at codon 242 (pS242I). The mechanisms by which these mutations in *ZNF687* affect bone metabolism to cause PDB and GCT remain unclear. Expression of *ZNF687* increases during osteoclastogenesis following M-CSF and RANKL treatment of human monocytes. The gene is also expressed in monocytes, osteoclasts, and osteoblasts in zebrafish, and high levels of *ZNF687* mRNA have also been found in GCT. While bioinformatic analysis has predicted that the pP937R mutation may enhance nuclear import of *ZNF687*, the consequences of this are unclear.

## Pharmacogenetics and PDB

The importance of genetic factors in PDB raises the possibility that genetic profiling might be of value clinically in identifying those at risk of the disease and also in providing markers of response to treatment. Both of these issues are discussed in the present section.

### Genetic Markers for Susceptibility to PDB

The importance of genetic factors in PDB coupled with the observation that risk variants have a large effect size raises the possibility that genetic testing could be of value in identifying patients at risk of developing PDB or those at risk of complications [91]. This is particularly relevant in the rare syndromic forms of PDB where the disease is inherited in a Mendelian manner where genetic testing is already performed in children of affected individuals. The potential role of genetic testing coupled to therapeutic intervention is currently being explored in the context of the ZiPP trial (ISRCTN 11616770). In this study, adults who had a family history of PDB were offered genetic testing for *SQSTM1* mutations, and those that tested positive were invited to take part in a randomised controlled trial of zoledronic acid or placebo with the aim of determining whether treatment was effective at preventing the development of PDB [92]. Analysis of baseline characteristics showed that about 9% of *SQSTM1* mutation carriers had signs of PDB on bone scan but were asymptomatic, and interestingly, most people with lesions had normal levels of biochemical markers of bone remodelling [93•]. The study remains in progress but is due to report during 2021. Another approach used by Guay-Belanger and colleagues combined both a genetic screening test for *SQSTM1* mutations, with a test for biochemical markers associated with PDB [94]. This approach correctly identified 93.9% of PDB-affected patients as compared with controls. Similarly, Albagha and colleagues evaluated the performance of seven SNPs associated with PDB derived from GWAS with mutations in *SQSTM1* to try and predict extent and severity of PDB in a multinational cohort of 1940 patients with the disease. The approach was successful in identifying disease extent, complications, and number of courses of bisphosphonates administered [91]. Both Guay-Belanger’s study and Albagha’s study focused on patients known to have PDB rather than those at risk because of positive family history, and a future aim will be to further explore the risk of genetic and other markers in predicting risk of developing the disease.

### Genetic Markers of Treatment Response

Several investigators have explored the possible role of genetic profiling in assessing treatment response [95–97]. Bisphosphonates are generally considered to be the treatment of first choice for PDB, and modern bisphosphonates can induce biochemical remission in the majority of PDB patients [98, 99]. Some investigators have studied so-called acquired resistance to bisphosphonate therapy in PDB, although these studies typically involved patients treated with older bisphosphonates [95, 100–102]. Mossetti and colleagues [96] studied the relation between the *TaqI*, *BsmI*, and *Fok1* polymorphisms in the *VDR* gene and response to clodronate in 84 patients with PDB as well as possible associations between *SQSTM1* mutations and treatment response in the same population. They reported a significant association between the *TaqI* and *BsmI* polymorphisms and resistance to 1500mg clodronate given intravenously by logistic regression analysis taking other predictors into account. Resistance was defined as lack of suppression of ALP level by more than 50% compared with the starting value. The duration of response was also related to *VDR* alleles. There was no difference however in the proportion of patients with *SQSTM1* mutations in the groups of patients with resistance or no resistance to clodronate. Corral-Gudino and colleagues [95] studied the relation between polymorphisms in the *IL1B*, *ILR1*, and *IL1RN* genes and response to treatment with various bisphosphonates including etidronate, tiludronate, clodronate, risedronate and pamidronate in 123 patients with PDB. The *IL1B* gene encodes interleukin-1 beta a cytokine with stimulatory effects on osteoclasts; the *ILR1* gene encodes the interleukin 1 receptor, and the *IL1RN* gene encodes the interleukin-1 receptor antagonist. They reported that the −511C/T polymorphism of the *IL1B* gene was significantly associated with response to all bisphosphonates grouped together [95]. Analysis of response to individual bisphosphonates revealed notionally significant results for tiludronate and risedronate, but the analyses were not corrected for multiple testing. The mechanisms of acquired resistance to bisphosphonate therapy in PDB remain poorly understood, but several theories are proposed. The first is that bisphosphonate therapy may select a subgroup of osteoclasts that become resistant to the apoptotic effect of the drug [103]. The second is that continued therapy with a bisphosphonate may induce enzymes that confer resistance to a subset of osteoclasts or their precursors [103]. It has also been proposed that acquired resistance is caused by disease-related factors and patients with extensive PDB are more likely to develop resistance. These studies, while of interest, were both based on small sample size, and the results will be required to be replicated in other populations. More recently Visconti looked at the relation between *SQSTM1* mutations and treatment response in the PRISM study [97]. While carriers of *SQSTM1* mutations were found to have more severe disease, there was no difference between those with and without *SQSTM1* mutations and response to treatment.

## Conclusion

Genetic factors play a key role in the pathogenesis of Paget’s disease, and huge advances have been made over the past 20 years in identifying the genetic variants that predispose to the disease and the mechanisms by which they affect bone metabolism. Further research needs to be conducted to identify additional genes that cause the disease however, and it is likely that extended GWAS studies, genome sequencing studies, and candidate gene studies may cast further light into disease mechanisms in the future. An important direction will be to further explore the mechanisms by which genetic variants and environmental factors interact to influence susceptibility and disease severity, since this is an area that is poorly understood. Advances in genetics offer the prospect of developing a more comprehensive understanding of the pathophysiology of PDB and open the possibility of using genetic testing coupled to targeted therapeutic intervention to prevent disease progression and improve outcome before irreversible skeletal damage has occurred.
